# Supplementary material for: Construction of a web-based nanomaterial database by big data curation and modeling friendly nanostructure annotations
Source: Nat Commun. 2020 May 20;11:2519. doi: 10.1038/s41467-020-16413-3 (PMC7239871; doi:10.1038/s41467-020-16413-3)
Supplement: Supplementary file 1 — Supplementary Information [file 41467_2020_16413_MOESM1_ESM.pdf]

Supplementary Information

Construction of a web-based nanomaterial database by big data curation and modeling friendly nanostructure annotations

Yan *et al.*

Supplementary references:

1. Wu, L. *et al.* Tuning cell autophagy by diversifying carbon nanotube surface chemistry. *ACS Nano* **8**, 2087–2099 (2014).
2. Zhou, H. *et al.* A nano-combinatorial library strategy for the discovery of nanotubes with reduced protein-binding, cytotoxicity, and immune response. *Nano Lett.* **8**, 859–865 (2008).
3. Sun, H. *et al.* Induction of oxidative stress and sensitization of cancer cells to paclitaxel by gold nanoparticles with different charge densities and hydrophobicities. *J. Mater. Chem. B* **6**, 1633–1639 (2018).
4. Zhang, Y. *et al.* Modulation of Carbon Nanotubes' Perturbation to the Metabolic Activity of CYP3A4 in the Liver. *Adv. Funct. Mater.* **26**, 841–850 (2016).
5. Liu, Y. *et al.* Elucidation of the Molecular Determinants for Optimal Perfluorooctanesulfonate Adsorption Using a Combinatorial Nanoparticle Library Approach. *Environ. Sci. Technol.* **51**, 7120–7127 (2017).
6. Wang, W. *et al.* Universal nanohydrophobicity predictions using virtual nanoparticle library. *J. Cheminform.* **11**, 6 (2019).
7. Zhang, B. *et al.* Functionalized carbon nanotubes specifically bind to α-chymotrypsin's catalytic site and regulate its enzymatic function. *Nano Lett.* **9**, 2280–2284 (2009).
8. Su, G. *et al.* Effective surface charge density determines the electrostatic attraction between nanoparticles and cells. *J. Phys. Chem. C* **116**, 4993–4998 (2012).

9. Liu, Y., Winkler, D. A., Epa, V. C., Zhang, B. & Yan, B. Probing enzyme-nanoparticle interactions using combinatorial gold nanoparticle libraries. *Nano Res.* **8**, 1293–1308 (2015).

10. Zhou, H., Jiao, P., Yang, L., Li, X. & Yan, B. Enhancing cell recognition by scrutinizing cell surfaces with a nanoparticle array. *J. Am. Chem. Soc.* **133**, 680–682 (2011).

11. Bai, X. *et al.* Regulation of cell uptake and cytotoxicity by nanoparticle core under the controlled shape, size, and surface chemistries. *ACS Nano* **14**, 289–302 (2020).

External data references:

1. Vigderman, L., Manna, P. & Zubarev, E. R. Quantitative replacement of cetyl trimethylammonium bromide by cationic thiol ligands on the surface of gold nanorods and their extremely large uptake by cancer cells. *Angew. Chemie - Int. Ed.* **51**, 636–641 (2012).

2. Elci, S. G. *et al.* Dual-Mode Mass Spectrometric Imaging for Determination of in Vivo Stability of Nanoparticle Monolayers. *ACS Nano* **11**, 7424–7430 (2017).

3. Liu, X., Jin, Q., Ji, Y. & Ji, J. Minimizing nonspecific phagocytic uptake of biocompatible gold nanoparticles with mixed charged zwitterionic surface modification. *J. Mater. Chem.* **22**, 1916–1927 (2012).

4. Cho, W. S. *et al.* Zeta potential and solubility to toxic ions as mechanisms of lung inflammation caused by metal/metal oxide nanoparticles. *Toxicol. Sci.*

126, 469–477 (2012).

5. You, C., De, M., Han, G. & Rotello, V. M. Tunable Inhibition and Denaturation of α-Chymotrypsin with Fabrication of Amino Acid-Functionalized Gold Nanoparticles. *J. Am. Chem. Soc.* **127**, 12873–12881 (2005).
6. Toropova, A. P., Toropov, A. A., Benfenati, E., Leszczynska, D. & Leszczynski, J. QSAR modeling of measured binding affinity for fullerene-based HIV-1 PR inhibitors by CORAL. *J. Math. Chem.* **48**, 959–987 (2010).
7. Jiang, Y. *et al.* The Interplay of Size and Surface Functionality on the Cellular Uptake of Sub-10 nm Gold Nanoparticles. *ACS Nano* **9**, 9986–9993 (2015).
8. Sato, M. *et al.* New surface-modified zinc oxide nanoparticles with aminotriethylene oxide chains linked by 1,2,3-triazole ring: Preparation, and visible light-emitting and noncytotoxic properties. *Appl. Surf. Sci.* **258**, 786–790 (2011).
9. Slone, S. M. Building DNA Brick Structures with LegoGen. 1–16 (2016).
10. Abdelhameed, M. *et al.* Tuning the Optical Properties of Silicon Quantum Dots via Surface Functionalization with Conjugated Aromatic Fluorophores. *Sci. Rep.* **8**, 1–10 (2018).
11. Hutter, E. *et al.* Microglial response to gold nanoparticles SUPPORTING INFO. *ACS Nano* **4**, 2595–2606 (2010).
12. Saha, K. *et al.* Regulation of Macrophage Recognition through the Interplay of Nanoparticle Surface Functionality and Protein Corona. *ACS Nano* **10**, 4421–

4430 (2016).

13. Liu, J. *et al.* Dependences of water permeation through cyclic octa-peptide nanotubes on channel length and membrane thickness. *J. Chem. Inf. Model.* **52**, 2132–2138 (2012).

14. Choi, A. O., Ju, S. J., Desbarats, J., Lovrić, J. & Maysinger, D. Quantum dot-induced cell death involves Fas upregulation and lipid peroxidation in human neuroblastoma cells. *J. Nanobiotechnology* **5**, 1–13 (2007).

15. Zhu, Z. J. *et al.* The interplay of monolayer structure and serum protein interactions on the cellular uptake of gold nanoparticles. *Small* **8**, 2659–2663 (2012).

16. Morilla, M., Schilrreff, Mundiña-Weilenmann & Romero. Selective cytotoxicity of PAMAM G5 core-PAMAM G2.5 shell tecto-dendrimers on melanoma cells. *Int. J. Nanomedicine.* **7**, 4121-4133 (2012)..

17. Choi, K. M., Kwon, C. H., Kim, H. L. & Hwang, H. Potential of mean force calculations for ion selectivity in a cyclic peptide nanotube. *Bull. Korean Chem. Soc.* **33**, 911–916 (2012).

18. Gold, A. A. *et al.* Surface Charge Density of Amino Acid- Efficient Gene Delivery Vectors by Tuning the Surface Charge Density of. *Society* **2**, 2213–2218 (2008).

19. Yoo, J., Li, C., Slone, S. M. & Maffeo, C. A Practical Guide to Molecular Dynamics Simulations of DNA Origami Systems. *DNA Nanotechnology. Hum. Press. New York, NY.* **5**, 209–229 (2018).

20. Bartneck, M. *et al.* Rapid uptake of gold nanorods by primary human blood phagocytes and chemistry. *ACS Nano* **4**, 3073–3086 (2010).

21. Shankar, S. & Rhim, J. W. Amino acid mediated synthesis of silver nanoparticles and preparation of antimicrobial agar/silver nanoparticles composite films. *Carbohyd. Polym.* **130**, 353–363 (2015).

22. Maingi, V., Jain, V., Bharatam, P. V. & Maiti, P. K. Dendrimer building toolkit: Model building and characterization of various dendrimer architectures. *J. Comput. Chem.* **33**, 1997–2011 (2012).

23. Huang, K. *et al.* Size-dependent localization and penetration of ultrasmall gold nanoparticles in cancer cells, multicellular spheroids, and tumors in vivo. *ACS Nano* **6**, 4483–4493 (2012).

24. Liu, R. *et al.* Classification NanoSAR development for cytotoxicity of metal oxide nanoparticles. *Small* **7**, 1118–1126 (2011).

25. Dong, Y., Chen, S., Zhang, S. & Sodroski, J. Folding DNA into a Lipid-Conjugated Nanobarrel for Controlled Reconstitution of Membrane Proteins. *Angew. Chem.* **130**, 2094–2098 (2018).

26. Elci, S. G. *et al.* Surface Charge Controls the Suborgan Biodistributions of Gold Nanoparticles. *ACS Nano* **10**, 5536–5542 (2016).

27. Abdelmonem, A. M. *et al.* Charge and agglomeration dependent in vitro uptake and cytotoxicity of zinc oxide nanoparticles. *J. Inorg. Biochem.* **153**, 334–338 (2015).

28. Goodman, C. M., McCusker, C. D., Yilmaz, T. & Rotello With, V. M. Toxicity

of GNPs functionalized Bioconjugate, cationic and anionic side chains. *Bioconjug. Chem.* **15**, 897–900 (2004).

29. Arvizo, R. R. *et al.* Modulating pharmacokinetics, tumor uptake and biodistribution by engineered nanoparticles. *PLoS One* **6**, 3–8 (2011).

30. Sumiya, N., Igami, D. & Takeda, K. Molecular dynamical study on ion channeling through peptide nanotube. *AIP Conf. Proc.* **1399**, 1055–1056 (2011).

31. Long, Y. M. *et al.* Surface ligand controls silver ion release of nanosilver and its antibacterial activity against Escherichia coli. *Int. J. Nanomedicine* **12**, 3193–3206 (2017).

32. Qiu, Y. *et al.* Surface chemistry and aspect ratio mediated cellular uptake of Au nanorods. *Biomaterials* **31**, 7606–7619 (2010).

33. Chompoosor, A. *et al*. The role of surface functionality on acute cytotoxicity, ROS generation and DNA damage by cationic gold nanoparticles. *Small* **6**, 2246–2249 (2010).

34. Nguyen, N. *et al.* The absence of tertiary interactions in a self-assembled DNA crystal structure. *J. Mol. Recognit*. **25**, 234–237 (2012).

35. Maiti, P. K., Çağin, T., Wang, G. & Goddard, W. A. Structure of PAMAM dendrimers: Generations 1 through 11. *Macromolecules* **37**, 6236–6254 (2004).

36. Si, X. *et al.* Dynamic behavior and selective adsorption of a methanol/water mixture inside a cyclic peptide nanotube. *J. Mol. Model.* **24**, (2018).

37. De, M. *et al.* Sensing of Proteins in Human Serum using Nanoparticle-Green

Fluorescent Protein Conjugates. *Nat. Chem.* **1**, 461–465 (2010).

38. Pallotta, A., Boudier, A., Leroy, P. & Clarot, I. Characterization and stability of gold nanoparticles depending on their surface chemistry: Contribution of capillary zone electrophoresis to a quality control. *J. Chromatogr. A* **1461**, 179–184 (2016).

39. Bozich, J. S. *et al.* Surface chemistry, charge and ligand type impact the toxicity of gold nanoparticles to Daphnia magna. *Environ. Sci. Nano* **1**, 260–270 (2014).

40. Moyano, D. F. *et al.* Fabrication of Corona Free Nanoparticles with Tunable Hydrophobicity. *ACS Nano* **8**, 6748–6755 (2014).

41. Zhu, Z. J. *et al.* Surface properties dictate uptake, distribution, excretion, and toxicity of nanoparticles in fish. *Small* **6**, 2261–2265 (2010).

42. Miranda, O. R. *et al.* Enzyme-amplified array sensing of proteins in solution and in biofluids. *J. Am. Chem. Soc.* **132**, 5285–5289 (2010).

43. Hu, D. *et al.* Surface-Adaptive Gold Nanoparticles with Effective Adherence and Enhanced Photothermal Ablation of Methicillin-Resistant Staphylococcus aureus Biofilm. *ACS Nano* **11**, 9330–9339 (2017).

44. Arvizo, R. R. *et al.* Effect of nanoparticle surface charge at the plasma membrane and beyond. *Nano Lett.* **10**, 2543–2548 (2010).

45. Porret, E. *et al.* Hydrophobicity of Gold Nanoclusters Influences Their Interactions with Biological Barriers. *Chem. Mater.* **29**, 7497–7506 (2017).

46. Bartczak, D. *et al.* Interactions of human endothelial cells with gold

nanoparticles of different morphologies. *Small* **8**, 122–130 (2012).

47. Bai, X., Martin, T. G., Scheres, S. H. W. & Dietz, H. Cryo-EM structure of a 3D DNA-origami object. *PNAS*. **109**, 20012-20017 (2012).

48. Boselli, L., Polo, E., Castagnola, V. & Dawson, K. A. Regimes of Biomolecular Ultrasmall Nanoparticle Interactions. *Angew. Chemie - Int. Ed.* **56**, 4215–4218 (2017).

49. Farrokhpour, H., Mansouri, A., Rajabi, A. R. & Najafi Chermahini, A. The effect of the diameter of cyclic peptide nanotube on its chirality discrimination. *J. Biomol. Struct. Dyn.* **37**, 691–701 (2019).

50. Cho, S. J. *et al.* Long-term exposure to CdTe quantum dots causes functional impairments in live cells. *Langmuir* **23**, 1974–1980 (2007).

51. Liu, J. *et al.* Water Diffusion Behaviors and Transportation Properties in Transmembrane Cyclic Hexa-, Octa- and Decapeptide Nanotubes. *J. Phys. Chem. B* **114**, 12183–12192 (2010).

52. Walkey, Carl; Olsen, Jonathan; Song, Fayi; Liu, Rong; Guo, Hongbo; Olsen, Wesley; Cohen, Yoram; Emili, Andrew; Chan, W. the Cellular Interaction of Gold and. *ACS Nano* **8**, 2439–2455 (2014).

53. Chang, S., Kang, B., Liu, X., Dai, Y. & Chen, D. The combined influence of surface modification, size distribution, and interaction time on the cytotoxicity of CdTe quantum dots in PANC-1 cells. *Acta Biochim Biophys Sin* **44**, 241–248 (2012).

54. Li, X. *et al.* Functional gold nanoparticles as potent antimicrobial agents

against multi-drug-resistant bacteria. *ACS Nano* **8**, 10682–10686 (2014).

55. Mizuhara, T. *et al.* Acylsulfonamide-Functionalized zwitterionic gold nanoparticles for enhanced cellular uptake at tumor pH. *Angew. Chemie - Int. Ed.* **54**, 6567–6570 (2015).

56. Pan, K. *et al.* Lattice-free prediction of three-dimensional structure of programmed DNA assemblies. *Nat. Commun.* **5**, 5578 (2014).

57. Moyano, D. F. *et al.* Nanoparticle hydrophobicity dictates immune response. *J. Am. Chem. Soc.* **134**, 3965–3967 (2012).

58. Zhang, H. *et al.* Use of metal oxide nanoparticle band gap to develop a predictive paradigm for oxidative stress and acute pulmonary inflammation. *ACS Nano* **6**, 4349–4368 (2012).

59. Nagy, A. *et al.* Comprehensive analysis of the effects of CdSe quantum dot size, surface charge, and functionalization on primary human lung cells. *ACS Nano* **6**, 4748–4762 (2012).

60. Yang, X. *et al.* Pharmaceutical Intermediate-Modified Gold Nanoparticles: Against Multidrug-Resistant Bacteria and Wound-Healing Application via an Electrospun Scaffold. *ACS Nano* **11**, 5737–5745 (2017).

61. Huo, S. *et al.* Fully Zwitterionic Nanoparticle Antimicrobial Agents through Tuning of Core Size and Ligand Structure. *ACS Nano* **10**, 8732–8737 (2016).

62. Byrne-Nash, R. *et al.* Probing the Mechanism of LAL-32, a Gold Nanoparticle-Based Antibiotic Discovered through Small Molecule Variable Ligand Display. *Bioconjug. Chem.* **28**, 1807–1810 (2017).

63. Naha, P. C., Davoren, M., Lyng, F. M. & Byrne, H. J. Reactive oxygen species (ROS) induced cytokine production and cytotoxicity of PAMAM dendrimers in J774A.1 cells. *Toxicol. Appl. Pharmacol.* **246**, 91–99 (2010).

64. Prasad, B. R. *et al.* Effects of long-term exposure of gelatinated and non-gelatinated cadmium telluride quantum dots on differentiated PC12 cells. *J. Nanobiotechnology* **10**, 1–14 (2012).

65. Dreaden, E. C. *et al.* Small molecule-gold nanorod conjugates selectively target and induce macrophage cytotoxicity towards breast cancer cells. *Small* **8**, 2819–2822 (2012).

66. Zhao, Y. *et al.* Small molecule-capped gold nanoparticles as potent antibacterial agents that target gram-negative bacteria. *J. Am. Chem. Soc.* **132**, 12349–12356 (2010).

67. Giri, K. *et al.* Targeting bacterial biofilms via surface engineering of gold nanoparticles. *RSC Adv.* **5**, 105551–105559 (2015).

68. Carvajal-Diaz, J. A. & Cagin, T. Electrophoretic Transport of Na+ and K+ Ions Within Cyclic Peptide Nanotubes. *J. Phys. Chem. B* **120**, 7872–7879 (2016).

69. Niikura, K.; Matsunaga, T.; Suzuki, T.; Kobayashi, K.; Yamaguchi, H.; Orba, Y.; Kawaguchi, A.; Hasegawa, H; Kajino, K.; Ninomiya, T.; Ijiro, K.; Hirofumi, S. Gold nanoparticles as a vaccine platform: influence of size and shape on immunological responses in vitro and in vivo. *ACS Nano* **7**, 3926–3938 (2013).
