# Supplementary material for: Construction of a web-based nanomaterial database by big data curation and modeling friendly nanostructure annotations
Source: Nat Commun. 2020 May 20;11:2519. doi: 10.1038/s41467-020-16413-3 (PMC7239871; doi:10.1038/s41467-020-16413-3)
Supplement: Supplementary file 3 — Description of Additional Supplementary Files [file 41467_2020_16413_MOESM3_ESM.pdf]

Description of Additional Supplementary Files

File name: Supplementary Data 1
Description: Structure and biological data with detailed experimental protocol information
